# Supplementary material for: No tears in heaven: did the media create the pseudo-phenomenon “altitude-adjusted lachrymosity syndrome (AALS)”?
Source: PeerJ. 2018 Apr 3;6:e4569. doi: 10.7717/peerj.4569 (PMC5888120; doi:10.7717/peerj.4569)
Supplement: Appendix S1 — Grey Literature Review Summary Table. [file peerj-06-4569-s003.docx]

Appendix One: Concept elicitation using Google Search terms

Search term: crying on planes (no quotation marks) on Google.com in July 2017

| **Hit** | **Title** | **Relevant?** | **Domains Mentioned** | **Possible Causes Posited** | **URL** |
| --- | --- | --- | --- | --- | --- |
| 1 | Wolfson E (2013) Why We Cry on Planes, The Atlantic, October 1, 2013 | Yes | Crying during non-sad film (Miss Congeniality)  Guilty pleasure films | Solitude  Altitude  Lack of distraction  Fear of death  Separation  Rejection  “Down time”  Loss of control  Life events | https://www.theatlantic.com/health/archive/2013/10/why-we-cry-on-planes/280143/ |
| 2 | Anwar M (2015) Why do people cry more on airplanes? Here’s the science behind those airborne tears, Bustle.com, November 23, 2015 | Yes | Crying at films on planes | Evolutionary biology  Eliciting sympathy  Anxiety  Loneliness  Helplessness  Lack of distraction  Boredom | https://www.bustle.com/articles/125640-why-do-people-cry-more-on-airplanes-heres-the-science-behind-those-airborne-tears |
| 3 | Lewis A (2017) Why do people cry more on planes?, Tonic.Vice.com, February 13, 2017 | Yes | Crying during non-sad film (Miss Congeniality 2, the Lobster) | Watching particularly emotional films (e.g. Inside Out, Steve Jobs)  Anxiety  Loss of control  Lower oxygen levels  Lack of sleep  Life events  Lack of distraction | https://tonic.vice.com/en_us/article/8qk93p/why-do-people-cry-more-on-planes |
| 4 | Ubaldi S (2016) Why do we always cry on planes?, AWOL.junkee.com, November 4, 2016 | Yes | Crying excessively during sad films (Still Alice, The Impossible, Foxcatcher)  Crying during non-sad film (How to be single) | Being tired  Life events  Lower oxygen levels  Lower cabin pressure  “Down time”  Solitude  Perceived privacy  Engine noise  Adrenal cortisol | https://awol.junkee.com/why-do-we-always-cry-on-planes/36056 |
| 5 | McBain S (2016) Tears in Heaven: Why do we cry on aeroplanes?, New Statesman, July 21, 2016 | Yes | Crying during non-sad film (Bridesmaids, The Simpsons, Pitch Perfect 2) | Watching particularly emotional films (tearjerkers)  Life events  Anxiety  Powerlessness  Solitude  Reflecting on feelings in private  Lack of distraction  Empathizing with film characters  Proximity to screen  Feeling like a child  Altitude  Feeling tired  Alcohol  Oxygen levels  Cabin pressure  Loneliness | http://www.newstatesman.com/politics/uk/2016/07/tears-heaven-why-do-we-cry-aeroplanes |
| 6 | McBain S (2016) Tears in Heaven: Why do we cry on aeroplanes?, Financial Review, August 24, 2016 | Repost of above | Repost of 5 above | Repost of above | http://www.afr.com/lifestyle/travel/tears-in-heaven-why-do-we-cry-on-aeroplanes-20160807-gqn8u8 |
| 7 | Walansky A (2017) What’s the proper etiquette for dealing with crying babies on a plane?, Bravo.com Jet Set, January 18, 2017 | No | Not relevant | Not relevant | http://www.bravotv.com/blogs/whats-the-proper-etiquette-for-dealing-with-crying-babies-on-a-plane |
| 8 | Anonymous Commenters (2014) Talking point: Why do we cry on planes?, The Guardian, October 8, 2014 | Yes | Crying excessively during sad films (Up, Precious, PS I Love You, Sunshine on Leath, Made in Dagenham, Kannathil Muthamittal)  Crying during non-sad film (True Romance, Rocky Balboa) | Watching particularly emotional films (tearjerkers)  Boredom  Feeling tired  Alcohol  Cabin pressure  Elevated CO2 levels  Reflecting on feelings in private  Anxiety  Passivity  Life events  Catharsis  Seeing your children  Nostalgia  Fear of death  Vulnerability  Isolation  “Aeroplane headaches”  Food quality  Helplessness  Discomfort  Boredom | https://www.theguardian.com/travel/2014/oct/08/why-do-we-cry-on-planes |
| 9 | MacMillen H (2017) Why I Love Crying on Airplanes, Allure.com, July 6, 2017 | Yes | Silent sobbing | Bereavement  Reflecting on feelings in private  Lack of distraction  Solitude  Feeling tired  Oxygen levels  Alcohol  Perceived privacy  Catharsis  Lack of judgement | https://www.allure.com/story/why-i-love-crying-on-airplanes |
| 10 | Klass P (2016) Fear of Crying: The Problem of Babies and Airplanes | No | Not relevant | Not relevant | https://well.blogs.nytimes.com/2016/04/11/fear-of-crying-the-problem-of-babies-and-airplanes/?_r=0 |
| 11 | Bourdet (2013) Why DO We Cry on Airplanes, Anyway?, Refinery29.com, October 2, 2013 | Yes | Crying at films on planes | “Down time”  Lack of distraction | http://www.refinery29.com/2013/10/54622/cry-airplanes |
| 12 | Coad J (2014) Why watching soppy films on planes makes men bawl like babies, The Daily Mail, August 12, 2014 | Yes | Reference to a survey conducted by Virgin Atlantic | Lack of sleep  Stress  “Down time”  Reflecting on feelings in private  Parasympathetic nervous system activation  Anxiety  Loss of control | http://www.dailymail.co.uk/health/article-2722466/Why-watching-soppy-films-planes-makes-men-bawl-like-babies.html |
| 13 | Anonymous (2014) Sky-High Sobbing: Why Men Cry During Movies on Planes | Yes | News segment  Crying excessively during sad films (Toy Story 3, Eat Pray Love, Saving Private Ryan)  Crying during non-sad film (The Hangover)  Using blankets to hide tears | Vulnerability  Alcohol | http://www.today.com/health/sky-high-sobbing-why-men-cry-during-movies-planes-1D80064709 |
| 14 | Cirilli C (2015) How to stop a baby from crying on a plane | No | Not relevant | Not relevant | http://www.foxnews.com/travel/2015/10/29/how-to-stop-baby-from-crying-on-plane.html |
| 15 | Schweitzer K (2016) This Video Will Completely Change your Attitude Toward That Crying Baby On Your Next Flight, Popsugar.co.uk, November 23, 2016 | No | Not relevant | Not relevant | https://www.popsugar.co.uk/smart-living/JetBlue-FlyBabies-Campaign-Crying-Babies-Planes-42754517?utm_medium=redirect&utm_campaign=US:GB&utm_source=www.google.com |
| 16 | Shortsleeve C (2016) 5 Ways to Keep Your Baby from Crying On Planes, CNTraveler.com, May 6, 2016 | No | Not relevant | Not relevant | http://www.cntraveler.com/stories/2016-05-05/5-ways-to-keep-your-baby-from-crying-on-planes |
| 17 | Anonymous (2016) Mum Invents Device to Stop Babies Crying On Planes, Cozigo.com, October 13, 2016 | No | Not relevant | Not relevant | https://cozigo.com/blogs/news/mum-invents-device-to-stop-babies-crying-on-planes |
| 18 | Anonymous (2017) Are there inventions/ways to stop babies from crying on planes/public spaces? Quora.com, accessed July 25, 2017 | No | Not relevant | Not relevant | https://www.quora.com/Are-there-inventions-ways-to-stop-babies-from-crying-on-planes-public-spaces |
| 19 | Cuskelly C (2017) THIS is why you cry more on planes and what one airline is doing to STOP it, Daily Express, July 7, 2017 | Yes | Introduction of “weepiness” warnings by Virgin Atlantic before sad films | Life events  Isolation  Loneliness  Oxygen levels  Air pressure  Being in economy | http://www.express.co.uk/travel/articles/825951/flights-cry-more-planes-emotional-warnings |
| 20 | Shetty K (2017) From crying in planes to losing his virginity when he was 13, GoT’s Kit Harrington gets candid!, Pinkvilla.com, February 2, 2017 | No | Not relevant | Not relevant | https://www.pinkvilla.com/entertainment/news/371850/crying-planes-losing-his-virginity-when-he-was-13-gots-kit-harington-gets |
