# Supplementary material for: No tears in heaven: did the media create the pseudo-phenomenon “altitude-adjusted lachrymosity syndrome (AALS)”?
Source: PeerJ. 2018 Apr 3;6:e4569. doi: 10.7717/peerj.4569 (PMC5888120; doi:10.7717/peerj.4569)
Supplement: Appendix S2 — Survey questionnaire. [file peerj-06-4569-s004.docx]

Supplementary Appendix Two: Survey fielded to participants

Survey:

**SCREENER**

1. When is the last time you watched a film on a plane?
   1. In the past 12 months
   2. More than 12 months ago <**Screen out**>
   3. Never <**Screen out**>

**ABOUT THE FILM ON A PLANE**

1. What was the name of the last film that you watched on a plane?
   1. (Open text)
2. How would you describe the category of this film? Please choose the one that *best* describes the film overall.
   1. Action
   2. Adventure
   3. Animated
   4. Biography
   5. Comedy
   6. Crime
   7. Documentary
   8. Drama
   9. Family
   10. Fantasy
   11. Film-Noir
   12. History
   13. Horror
   14. Music
   15. Musical
   16. Mystery
   17. Romance
   18. Romantic comedy
   19. Science Fiction
   20. Sport
   21. Thriller
   22. War
   23. Western
3. How would you rate this film on a scale of 0-10?
   1. 10 star scale

**IN-FLIGHT CRYING**

1. While watching this film, how often did you find yourself crying very easily?
   1. Never
   2. Rarely
   3. Occasionally
   4. Frequently
   5. Most of the time
2. While watching this film, how often did you find it hard to control your crying or urge to cry?
3. Never
4. Rarely
5. Occasionally
6. Frequently
7. Most of the time
8. Were you surprised at how much you cried or felt like crying at this film?
9. Yes
10. No

**IN-FLIGHT CRYING RISK FACTORS**

1. Had you recently had a particularly emotional or sentimental experience such as a wedding, a funeral, a reunion, or moving home?
2. Yes
3. No
4. Was this film a “guilty pleasure”, i.e. one you would normally be embarrassed to watch?
   1. Yes
   2. No
5. Had you consumed any alcohol during or just prior to watching this film?
   1. Yes, consumed some alcohol
   2. No, consumed no alcohol
6. When you watched the film were you feeling tired or jetlagged?
   1. Yes
   2. No
7. When you watched the film were you living with any medical, psychiatric, or neurological condition that you think affected your emotional state?
8. Yes (specify in open text)
9. No
10. (Optional) Is there anything else you want to add about your emotional reaction to watching this film?
11. (Open text)

**SCREENER FOR A GROUND FILM SINCE**

1. Since the film on the plane, have you watched another film when not on a plane?
   1. (If no, go to demographics)
   2. Yes, on television
   3. Yes, at the cinema
   4. Yes, on a computer, tablet, or smartphone

**THE LAST MOVIE YOU SAW THAT WAS NOT ON A PLANE**

1. What was the name of the most recent film that you watched that was not on a plane?
   1. (Open text)
2. How would you describe the category of this film? Please choose the one that best describes the film overall.
3. Action
4. Adventure
5. Animated
6. Biography
7. Comedy
8. Crime
9. Documentary
10. Drama
11. Family
12. Fantasy
13. Film-Noir
14. History
15. Horror
16. Music
17. Musical
18. Mystery
19. Romance
20. Romantic comedy
21. Science Fiction
22. Sport
23. Thriller
24. War
25. Western
26. How would you rate this film on a scale of 0-10?
27. 10 star scale

**GROUND FILM CRYING**

1. While watching this film, how often did you find yourself crying very easily?
   1. Never
   2. Rarely
   3. Occasionally
   4. Frequently
   5. Most of the time
2. While watching this film, how often did you find it hard to control your crying or urge to cry?
3. Never
4. Rarely
5. Occasionally
6. Frequently
7. Most of the time
8. Were you surprised at how much you cried or felt like crying at this film?
9. Yes
10. No

**GROUND FILM CRYING RISK FACTORS**

1. Had you recently had a particularly emotional or sentimental experience such as a wedding, a funeral, a reunion, or moving home?
   1. Yes
   2. No
2. Was this film a “guilty pleasure”, i.e. one you would normally be embarrassed to watch?
3. Yes
4. No
5. Had you consumed any alcohol during or just prior to watching this film?
6. Yes, consumed some alcohol
7. No, consumed no alcohol
8. When you watched the film were you feeling tired or jetlagged?
9. Yes
10. No
11. When you watched the film were you living with any medical, psychiatric, or neurological condition that you think affected your emotional state?
12. Yes (specify in open text)
13. No
14. (Optional) Is there anything else you want to add about your emotional reaction to watching this film?
15. (Open text)

**DEMOGRAPHICS**

1. How much total combined money did all members of your HOUSEHOLD earn last year?
   1. Prefer not to answer
   2. $0 to $9,999
   3. $10,000 to $24,999
   4. $25,000 to $49,999
   5. $50,000 to $74,999
   6. $75,000 to $99,999
   7. $100,000 to $124,999
   8. $125,000 to $149,999
   9. $150,000 to $174,999
   10. $175,000 to $199,999
   11. $200,000 and up
2. Are you male or female?
3. Male
4. Female
5. How old are you in years?
6. (Number)
7. What is your Mechanical Turk worker ID? You can find this on your dashboard at the top right
8. Open text
9. Enter this completion code into the MTurk Box where requested: BXCPD6TMGM
10. Yes I have entered this code into the MTurk Box

**<SUBMIT>**
